# Geographic disparities in telemedicine mental health use by applying three way ANOVA on Medicaid claims population data

**DOI:** 10.1186/s12913-024-10898-0

**Published:** 2024-04-22

**Authors:** Benjamin Ukert, Mark Lawley, Hye-Chung Kum

**Affiliations:** 1 Department of Health Policy and Management, Texas A&M University, College Station, TX USA; 2 Department of Industrial and Systems Engineering, Texas A&M University, College Station, TX USA; 3 Population Informatics Lab, Department of Health Policy and Management, Texas A&M University, College Station, TX USA

**Keywords:** Telemedicine, Healthcare access, Mental health, Health disparities

## Abstract

**Background:**

Utilization of telemedicine care for vulnerable and low income populations, especially individuals with mental health conditions, is not well understood. The goal is to describe the utilization and regional disparities of telehealth care by mental health status in Texas. Texas Medicaid claims data were analyzed from September 1, 2012, to August 31, 2018 for Medicaid patients enrolled due to a disability.

**Methods:**

We analyzed the growth in telemedicine care based on urban, suburban, and rural, and mental health status. We used t-tests to test for differences in sociodemographic characteristics across patients and performed a three-way Analyses of Variance (ANOVA) to evaluate whether the growth rates from 2013 to 2018 were different based on geography and patient type. We then estimated patient level multivariable ordinary least square regression models to estimate the relationship between the use of telemedicine and patient characteristics in 2013 and separately in 2018. Outcome was a binary variable of telemedicine use or not. Independent variables of interest include geography, age, gender, race, ethnicity, plan type, Medicare eligibility, diagnosed mental health condition, and ECI score.

**Results:**

Overall, Medicaid patients with a telemedicine visit grew at 81%, with rural patients growing the fastest (181%). Patients with a telemedicine visit for a mental health condition grew by 77%. Telemedicine patients with mental health diagnoses tended to have 2 to 3 more visits per year compared to non-telemedicine patients with mental health diagnoses. In 2013, multivariable regressions display that urban and suburban patients, those that had a mental health diagnosis were more likely to use telemedicine, while patients that were younger, women, Hispanics, and those dual eligible were less likely to use telemedicine. By 2018, urban and suburban patients were less likely to use telemedicine.

**Conclusions:**

Growth in telemedicine care was strong in urban and rural areas between 2013 and 2018 even before the COVID-19 pandemic. Those with a mental health condition who received telemedicine care had a higher number of total mental health visits compared to those without telemedicine care. These findings hold across all geographic groups and suggest that mental health telemedicine visits did not substitute for face-to-face mental health visits.

**Supplementary Information:**

The online version contains supplementary material available at 10.1186/s12913-024-10898-0.

## Background

Healthcare services delivered through remote technology, known as telemedicine, have been growing across the United States even before the COVID-19 pandemic [1–4]. Telemedicine presents itself to be a cost-effective alternative to the traditional face-to-face consultations between providers and patients [5–9]. Benefits may include greater access to care, more timely diagnoses of conditions, and the potential to close gaps in care. Telemedicine can be especially beneficial for individuals facing systemic barriers to access, such as those living in provider shortage areas or those with limited transportation options [10, 11].

The sudden universal reliance on telemedicine for medical care due to the COVID-19 pandemic has made it even more important to understand the conditions under which telemedicine is best used relying on available population data on telemedicine utilization. A detailed understanding of telemedicine utilization patterns before the COVID-19 pandemic will be critical to develop a good baseline. Innovative solutions to expand telemedicine delivery exists at the state level for those enrolled in Medicaid plans. States control Medicaid benefit packages and they have the option to include telemedicine as a mode of service delivery [12].

In Texas, Medicaid began covering telemedicine services in 1997 and telemedicine can be an especially promising tool of care delivery in Texas due to its size, extensive rural areas, and spatial disparities in access to care. Similarly, Texas has a large Medicaid population currently hovering close to 5 million patients, and covers a variety of residents with high disease burdens that may be especially suitable for telemedicine [13, 14]. Presently, over 400,000 disability-related individuals are covered by Texas Medicaid, representing mostly individuals with limitations that can onset at younger ages and people who are limited in walking or moving and taking care of personal need. Texas eligibility for disability coverage through Medicaid is determined based on the diagnosis of a disability by medical professionals that suggest the inability to engage in gainful activity (causes include blindness, accidents and other developed diseases) or the presence of mental impairment that lasts for more than a year, as described in 42 U.S.C. § 1382c(a)(3) [15]. Mental illness can be one factor that impairs functioning and independent living. It is common in the Medicaid population, with Medicaid being the largest payor for mental health services [16]. However, few people with mental illness receive mental health care treatment and little is known about the level of treatment in the Medicaid population and through telemedicine delivery specifically [1, 17, 18].

Understanding how Medicaid patients may be able to benefit from telemedicine, especially those with disabilities and mental illness, is important to reduce gaps in care that can otherwise lead to unnecessary acute care needs [19]. Further, little is known about the extent of mental health care and telemedicine visit growth in urban, suburban, and rural areas. Understanding growth patterns of telemedicine and mental health care delivery by geography is important for Texas and beyond [1, 17]. Additionally, there is a dearth of evidence on whether those with a mental illness are accessing mental telemedicine services in Texas.

To fill this knowledge gap, we describe the growth in telemedicine across rural and urban counties in Texas among Medicaid patients from 2013 to 2018. We focus on Medicaid patients with the highest potential need in Texas, those who are covered by Medicaid due to a disability, and then evaluate the use of mental health care and telemedicine-delivered mental health care for those diagnosed with a mental health diagnosis. Finally, we are also interested in evaluating whether the growth of telemedicine has led to more telemedicine mental health care visits that could have substituted for face-to-face mental healthcare visits. We do so by comparing trends in mental health use for disabled Medicaid patients diagnosed with a mental illness who engaged in telemedicine mental health compared to those that did not.

## Methods

Medicaid claims data for Medicaid Fee for Service (FFS) and Managed Care Organization (MCO) patients were obtained from Texas Health and Human Services Commission (HHSC). FFS Medicaid was the most common approach where the state pays providers based on the number and type of services rendered. Following national trends, Texas transitioned individuals from FFS to MCO health plans in the last 20 years and most individuals are enrolled in a MCO health plan today. In MCO plans the state pays a monthly fixed capitated payment for each covered individual to an insurance company who is then responsible for the management, delivery, and payment of healthcare services [20].

The data include all inpatient and outpatient care interactions and patient demographics, plan enrollment, and eligibility information. The claims included claims data from September 1, 2012 to August 31, 2018 (reflecting the collection of data for six state fiscal years) for Medicaid patients who had at least one healthcare interaction with a provider who billed for at least one telemedicine service procedure in a given state fiscal year. This adjusts for the fact that the number of providers providing telemedicine in these earlier years changed each year. Thus, the general study population are Medicaid patients who had access to a telemedicine provider in any given state fiscal year. We then restricted the dataset to those who were enrolled in a Medicaid plan under the disabled category because this group often faces barriers to in-person care and generally has behavioral health needs for which telemedicine may be especially suitable [21].

We define “patient” to refer to a disabled Medicaid patient whose provider engaged in some form of telemedicine; the term “tele patient” refers to a disabled Medicaid patient who used telemedicine; the term “mental health patient” refers to a disabled Medicaid patient who had a primary diagnosis of schizophrenia, bipolar disorder or depression (see Appendix Table 1 for diagnoses codes used) during a provider visit; and the term “mental health tele patient” refers to a disabled mental health patient who had a primary diagnosis for mental health during the telemedicine visit. We limit our analysis to those disabled Medicaid patients diagnosed with of schizophrenia, bipolar disorder or depression because we aim to focus on the disabled population who (1) have a diagnosed and long-term need for mental healthcare, and (2) where telemedicine represents an opportunity for continuous care delivery [2].

We examined growth in total patients, tele patients, mental health tele patients, volume of mental health visits, and mental health telemedicine visits for state fiscal years 2013–2018. Patients were identified based on whether they had a real-time interactive audio and video telemedicine visit or saw a provider who delivered telemedicine (but did not have a telemedicine visit). In some instances, it is possible that telemedicine was only rendered through audio, though, specific CPT and HCPCS codes for audio-only telemedicine visits became available for reimbursement purposes generally after 2017. Telemedicine visits were identified based on HCPCS procedure codes that directly identify teleservices (e.g. G0406-G0408) and CPT codes (e.g. 90,791–90,792) that included modifier codes GT or 95. A full list of codes can be found in Appendix Table 1. We identified mental health visits based on whether the primary diagnosis code included a diagnosis of schizophrenia, bipolar disorder or depression [22]. Diagnoses were identified from ICD9 and ICD10 codes.

Enrollment characteristics included age in years, gender, race (non-Hispanic white, non-Hispanic black, Hispanic, other), dual eligibility with Medicare, and FFS or MCO plan enrollment at the first time the patient was observed in the data in a state fiscal year. Patients were not required to be enrolled for the entire study period. Patients clinical profile was also described with the Elixhauser Comorbidity Score measured in the six months prior to the first of the telemedicine visit or the six months prior to the first eligibility month in a state fiscal year for non-telemedicine patients.

We combined patient’s county of residence with the 2013 nine county level urban-rural continuum information collected by the United States Department of Agriculture [23]. We defined urban counties as those with a metro area population of at least 250,000; suburban counties as those with an urban population of at least 2,500 and metro area population of less than 250,000; and rural counties as counties completely rural or having less than a 2,500 urban population.

The goal was to examine the growth across urban, suburban, and rural counties from 2013 to 2018 in the number of (1) Medicaid patients receiving any care from providers who deliver telemedicine, (2) tele patients, (3) mental health patients, and (4) mental health tele patients.

### Statistical analysis

We describe our sample by first presenting demographic characteristics for all patients and then separately for tele and non-tele patients. We used t-tests to test for differences in sociodemographic characteristics across patients. We then estimated patient level multivariable ordinary least square regression models to estimate the relationship between the use of telemedicine and patient characteristics in 2013 and separately in 2018. Variables of interest include geography, age, gender, race, ethnicity, plan type, Medicare eligibility, diagnosed mental health condition, and ECI score. Marginal effects are presented. We also estimate ordinary least square regression results with the dependent variable being the annual growth of patients, and the growth in the annual number of visits for telemedicine patients.

To describe changes in overall growth in patients, we used three-way Analyses of Variance (ANOVA) to evaluate whether the growth rates in patients from 2013 to 2018 were different based on geography and patient type. Specifically, we tested whether the growth rate was different by geography (urban vs. suburban vs. rural), tele vs. non-tele patients, and mental health vs. non-mental health patients. We also examine the interactions between these groups and settings and report the p-values relative to the rural counties when multiple comparisons were possible. The growth rate for each combination was computed as (total number of 2018 patients – total number of 2013 patients) / total number of 2013 patients. We then took the log transformation of these rates and applied the standard three-way ANOVA, using the 3-way interaction as an estimate of the mean squared error.

Finally, we also estimated the 2013 to 2018 relationship of annual growth rates and patient characteristics for the growth rate for patients with a mental health diagnosis vs. without a mental health diagnosis, the growth rate of patients who used telemedicine vs. non-telemedicine patients, and the growth rate of telemedicine use among patients with a mental health diagnosis vs. those patients with a mental health diagnosis without telemedicine use.

## Results

There were 519,454 patients in our disabled sample with 9.2% (48,024 patients) receiving some care through telemedicine (Table 1). The average age of the sample was 37.06 years (36.8 among tele patients, p-value = 0.002), 50.02% were female (47.0% among tele patients, *p* < 0.001), and 27.7% were dual eligible (23.8% among tele patients, *p* < 0.001). Among racial groups, 24.9% were non-Hispanic White (27.9% among tele patients, *p* < 0.001), 15.2% were non-Hispanic Black (14.7% among tele patients, *p* = 0.008), and 28% were Hispanic (27.1% among tele patients, *p* < 0.001). Finally, 45.2% of patients were enrolled in FFS Medicaid (35.6% among tele patients, *p* < 0.001), and 89.8% of patients lived in urban and suburban counties (86.9% among tele patients).

**Table 1:** Summary statistics of tele and non-tele health patients 2013–2018

	Total Sample	Telemedicine	Non-Telemedicine
Demographics			
Number of patients (%)	519,454 (100%)	48,024 (9.2%)	471,430 (91.8%)
Age	37.06	36.8	37.08†
Non-Hispanic White	24.9%	27.9%	24.6%†
Non-Hispanic Black	15.2%	14.7%	15.2%†
Hispanic	27.9%	27.1%	28.0%†
Women	50.2%	47.0%	50.5%†
Elixhauser Comorbidity Score	2.447	2.714	2.420†
Dual eligible	27.7%	23.8%	28.1%
Medicaid FFS	45.2%	35.6%	46.1%†
Urban	36.1%	33.2%	36.3%†
Suburban	53.7%	53.7%	53.7%
Rural	10.3%	13.1%	10.0%†
Trends in Mental Health and Telemedicine visits			
Average mental health visits per year	508,484	109,281	399,203
Average telemedicine visits per year	18,978	18,978	N/A
Average tele mental health visits per year	13,624	13,624	N/A
Number patients with mental health diagnosis	207,921	39,544	168,377

*Notes*: † indicates that the average is statistically different using t-test from the telemedicine patient mean at the 1% level

Regression results in Table 2 display that in 2013, urban and suburban patients were more likely than patients in rural counties to use telemedicine (Table 2, 2.7% points, *p* < 0.001; 5.8% points; *p* < 0.001, respectively). Patients that had a mental health diagnosis were more likely to use telemedicine (15.9% points, *p* < 0.001) while patients that were younger, women, Hispanics, and those dual eligible were less likely to use telemedicine. By 2018, urban and suburban patients were less likely than patients in rural counties to use telemedicine (4.9% points, *p* < 0.001; 3.5% points, *p* < 0.001). Patients that were younger, women, and those dual eligible were less likely to use telemedicine.

**Table 2:** Ordinary least square regressions of telemedicine use on patient characteristics 2013–2018

Telemedicine use	2013	2018
Urban	0.027†	-0.049†
Suburban	0.058†	-0.035†
Rural	Reference	Reference
Age	-0.001†	-0.001†
Women	-0.017†	-0.012†
Non-Hispanic White	-0.006	0.010†
Non-Hispanic Black	-0.007	-0.016†
Hispanic	-0.021†	0.017†
Medicaid FFS	-0.007	0.022†
Dual eligible	-0.041†	-0.024†
Mental Health Diagnosis	0.159†	0.158†
Elixhauser Comorbidity Score	0.001	0.003†

*Notes*: Coefficient estimates from ordinary least square patient-level regressions reported, where the dependent variable is a binary variable equal to one if patient used telemedicine services and zero otherwise. † indicates statistically significant coefficient at the 1% level

Of the 519,454 patients in the sample, 207,921 (40.0%) had a mental health diagnosis at a visit. Of the 48,024 tele patients, 39,544 (82.3%) had a mental health diagnosis, whereas among the 471,430 non-tele patients, only 168,377 (35.7%) had a mental health diagnosis. The 168,377 mental health non-tele patients generated an average of 399,203 mental health visits per year for an average of 2.37 visits per patient per year. While the 39,544 mental health tele patients generated an average of 109,281, mental health visits per year for an average of 2.76 visits per patient per year. On average, for mental health tele patients, 12.5% of mental health visits occurred via telemedicine across the 6-year period.

**Fig. 1:**
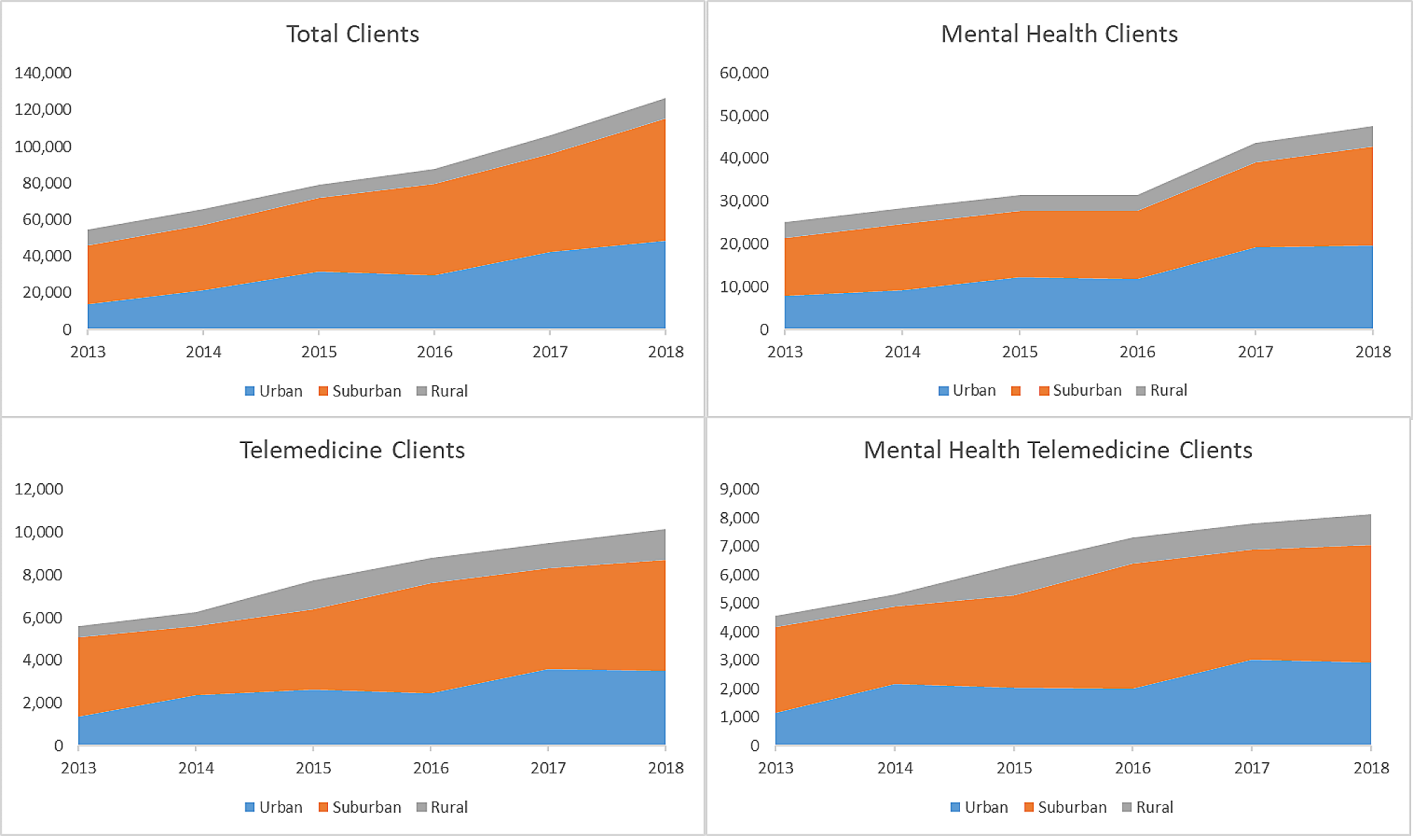
All patients, mental health patients, tele patients, and mental health tele patients. Notes: Figure describes the growth in the unique number of Medicaid patients seen by telemedicine providers in Texas

Figure 1 displays disabled Medicaid patient types and patient growth rate from year 2013 to 2018 for urban, suburban, and rural counties. The number of patients receiving care from a provider who engaged in some form of telemedicine grew from 54,455 to 126,580 (132%), with the largest growth rate being in urban patients (255%, *p* = 0.049). Over the same period, mental health patients grew by 90% from 25,177 (46% of total patients) to 47,719 (38% of total, *p* = 0.059), with urban mental health growth being the highest compared to the rural growth rate (152%, *p* = 0.124). See Appendix Table 2 for all counts.

The number of tele patients grew from 5,596 in 2013 to 10,145 in 2018 (81%, *p* = 0.132), with the largest growth rate being in rural patients (181%) followed closely by urban (152%, *p* = 0.779). Over the same period, the number of mental health tele patients grew from 4,582 (82% of all tele patients) to 8,138 (80% of tele patients, *p* = 0.190) with the largest growth rate being in rural counties (160%) followed closely by urban (155%). See also ordinary least squares multivariable regressions in Appendix Table 3. The percentage of tele patients represented 10% of all patients in 2013 to 8% in 2018. This trend was similar for urban (10–7%) and suburban (11–8%) but much different for rural areas, where the percentage of patients using telemedicine grew from 6% in 2013 to 13% in 2018. Similar results are seen for mental health patients, with urban tele mental health patients hovering around 15% of total mental health patients from 2013 to 2018, suburban tele mental health patients dropping from 22 to 18%, and rural tele mental health patients growing from 11 to 22%.

**Fig. 2:**
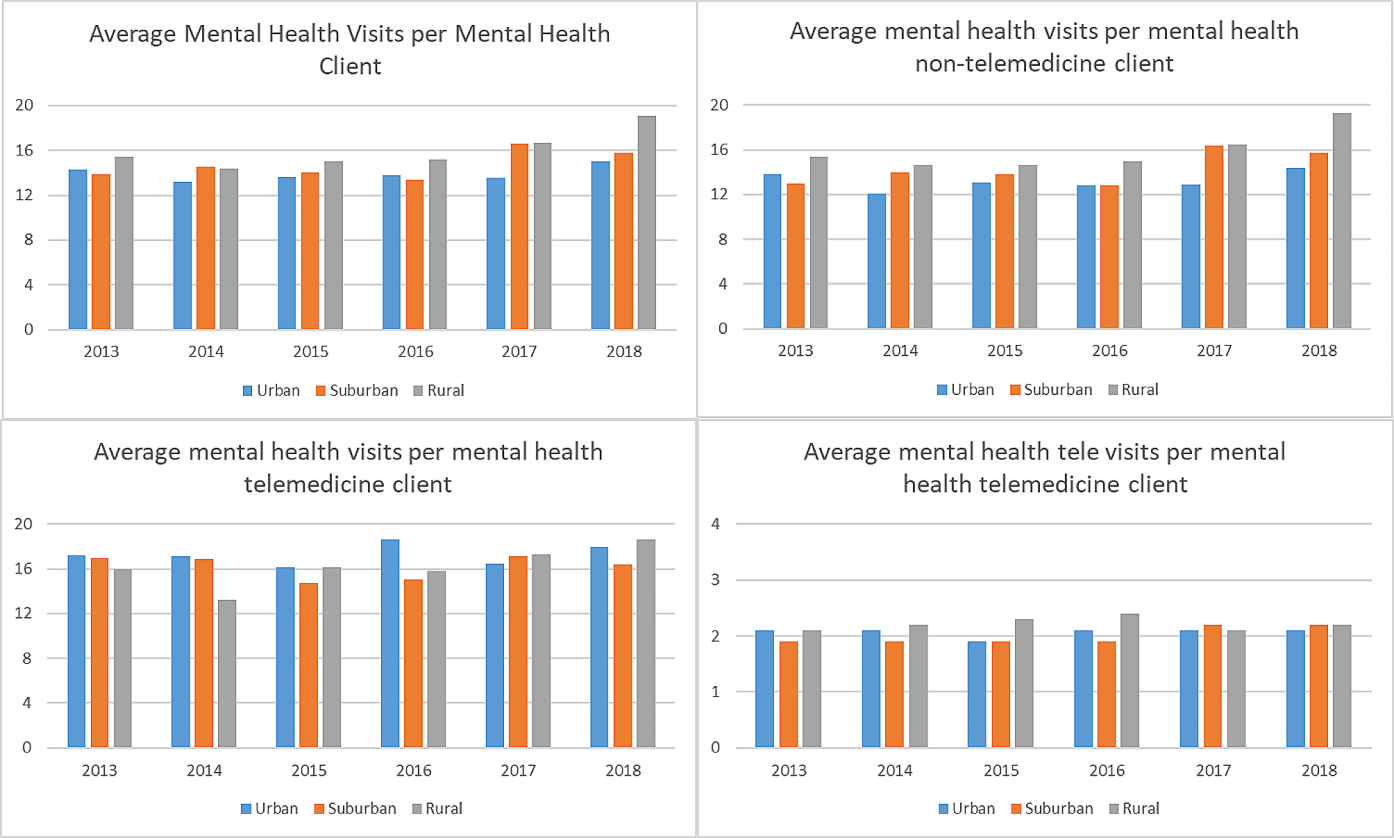
Average annual mental health visits per patient among mental health patients. Notes: Figure describes the average annual number of visits for different Medicaid patients

Figure 2 provides information on mental health visits. The average number of mental health visit per patient was about 14.5 per year for patients with a primary diagnosis of mental health during the visit. The average number of visits increased slightly from 2013 with 14.3 visits per patient per year to 15.8 visits per patient per year in 2018. Urban and suburban mental health visit rates were lower compared to rural mental health visit rates, with 14.3 and 13.9 visits per year in 2013 for urban and suburban patients and 15.4 visits per year in 2013 for rural patients. By 2018, all areas experienced growth in average visits compared to 2013, and rural mental health patients averaged 19.1 visits per year, while urban and suburban enrollees averaged 15 and 15.8 visits per year (*p* = 0.323 and *p* = 0.345, respectively). See counts in Appendix Table 4 and multivariable regressions in Appendix Table 5.

Stratifying mental health visits by telemedicine mental health and non-telemedicine mental health patients yields additional insights. Telemedicine mental health enrollees had, on average, 16.6 mental health visits compared to 14.0 mental health visits for non-telemedicine mental health patients (*p* < 0.001). Of note is that roughly two mental health visits out of the 16.6 mental health visits for telemedicine mental health patients were delivered through telemedicine. Thus, excluding telemedicine visits suggests that telemedicine mental health patients and non-telemedicine mental health patients had a similar level of mental health visits.

## Discussion

During the last 20 years, use in telemedicine accelerated, however, use varied substantially across geography and visit type. We observed an 81% growth rate of disabled Medicaid patients with a telemedicine visit between 2013 and 2018. However, patient panels grew at 132%, thus telemedicine usage did not keep pace with overall patient growth at telemedicine providers, with suburban patients showing the slowest increase. In contrast, for rural patients, telemedicine usage increased by 181% while the total number of rural patients increased by only 32%. Thus, relative to patient population size, the rural disabled Medicaid patients exhibited a much greater uptake of telemedicine than did the urban and suburban populations. Still, by 2018, 86% of telemedicine patients were still located in urban and suburban counties, which is similar to other published work, and large potential growth in telemedicine use persists as only 7% of all urban and suburban disabled Medicaid patients had a telemedicine visit [1, 24].

At the same time, the number of tele mental health patients increased by 77% from 2013 to 2018. As before, overall telemedicine usage among disabled Medicaid mental health patients did not keep pace with overall growth in mental health patients of 90%. This was due to the suburban population, which exhibited a 37% growth in tele mental health patients as opposed to a 77% growth in total mental health patients. The trend was just the opposite for rural counties, which exhibited a 75% growth rate in mental health patients and a 160% growth rate in tele mental health patients. Still, as of 2018, 87% of tele mental health patients were located in urban and suburban counties and only 16% of all urban and suburban disabled Medicaid patients with a mental health visit had a telemedicine mental health visit. Relative to earlier work that suggests relatively little mental healthcare delivery through telemedicine among the Medicare population; our findings paint a different and much more positive picture [2].

One of the most important observations on mental health visits is that telemedicine patients tended to have between two and three additional visits per year than non-telemedicine mental health patients. Given that the health risk profile looked similar across telemedicine groups, these findings hold across all geographic groups and suggest that mental health telemedicine visits may not substitute face-to-face visits, but that they could have led to additional face-to face visits. These findings are similar to work that has displayed that telemedicine care leads to additional care [9]. Whether additional visits may provide a positive return on investment for the disabled Medicaid population remains an open question.

Between 2013 and 2018, we also observed a shift in geography of disabled Medicaid patients seeing a telemedicine provider. The percentage living in urban, suburban, and rural counties shifted from 25%, 59%, 15% in 2013 to 38%, 53%, and 8% in 2018, respectively, reflecting a growing prevalence of disability in urban areas over this time, though we observe similar growth in telemedicine providers in urban and rural counties between 2013 and 2018. At the same time, we observed an 18% drop in the share of disabled patients with a mental health diagnosis. This was due to decreased prevalence of mental health diagnoses in suburban and rural populations, which fell by 26% and 46%, respectively. In contrast, the prevalence of mental health diagnoses in urban counties increased by 8%. This is in line with evidence that suggests that urban areas have greater access to mental health providers than rural areas [25]. Future growth in mental health telemedicine visits will depend on the availability and participation of providers. Still, by 2018, the majority of mental health patients (53%) were located in suburban counties.

The future policy direction of telemedicine care is evolving. The Center for Medicare and Medicaid Services (CMS) has been at the forefront of telemedicine delivery by establishing payments for physicians treating Medicare beneficiaries in 1999 [26]. However, Medicare provides reimbursement only to those living in rural areas and has only extended coverage temporarily during the COVID outbreak to all Medicare beneficiaries [12, 27]. At the onset of the COVID-19 pandemic, many providers shifted to provide telemedicine visits, and states Medicaid programs have expanded access to telemedicine use [13, 28]. State regulatory environment has pivoted back to pre COVID-19 policies, by for example, not allowing licensure waivers for telemedicine use across state borders [29].

Our study has limitations. First, while we believe we capture most video telemedicine visits, it is possible that some phone visits are included as nost audio-only CPT codes became available during the COVID-19 pandemic in 2020 and later. Second, our findings are limited to a subset of disabled patients who saw a telemedicine provider. Third, our findings for disabled Medicaid patients may not be generalizable to other populations, such as the disabled Medicaid population in other states and other non-disabled Medicaid patients. Fourth, telemedicine access requires that patients have IT equipment and broadband internet access. The large number of rural areas in Texas with potentially limited broadband access during this time may understate the growth in telemedicine delivery relative to other states. Finally, our data relies on data in a state with a long history of telemedicine reimbursement policies, thus one could expect that a larger share of providers is willing and able to deliver care through non-face to face means compared to other states.

## Conclusions

Our results have far-reaching implications for the future of telemedicine delivery in Texas and across the United States. The COVID-19 pandemic has led to substantial increases in short-term telemedicine use. Additionally, telemedicine has been championed to replace face-to-face visits. However, our study suggests that telemedicine care does not seem to substitute existing care among mental health visits, implying that additional care was delivered. Similar findings have previously been displayed among a sample of commercially insured employees [9]. However, telemedicine visits may have broader benefits, it could lead to improved continuity of care, early detection of condition on setting, and improve medication adherence. Most primary care physicians and specialists can agree that additional care seeking behavior may just display unmet needs, this is supported by the fact that the US has one of the lowest doctor visit ratios among OECD countries [30]. It remains to be seen whether telemedicine will remain a complement to care seeking rather than substituting care.

### Electronic supplementary material

Below is the link to the electronic supplementary material.

Supplementary Material 1

## Data Availability

The data that support the findings of this study are available from TX-HHSC but restrictions apply to the availability of these data due to confidentiality requirements. Data were used for evaluation purposes in the current study and so are not publicly available. Contact the corresponding author for more information on how to request the data.

## Abbreviations

ANOVA: Analyses of Variance
CMS: Center for Medicare and Medicaid Services
FFS: Medicaid Fee for Service
MCO: Managed Care Organization
HHSC: Texas Health and Human Services Commission
OECD: Organization for Economic Cooperation and Development
