# Supplementary material for: Geographic disparities in telemedicine mental health use by applying three way ANOVA on Medicaid claims population data
Source: BMC Health Serv Res. 2024 Apr 22;24:494. doi: 10.1186/s12913-024-10898-0 (PMC11034036; doi:10.1186/s12913-024-10898-0)
Supplement: Supplementary file 1 — Supplementary Material 1 [file 12913_2024_10898_MOESM1_ESM.docx]

**Appendix Tables**

**Appendix Table 1 – Code lists for Telemedicine and Mental Health Diagnoses**

| *Mental Health codes* | ICD9 | ICD10 |
| --- | --- | --- |
| Schizophrenia | 295* | F20*,F25* |
| Bipolar Disorder | 2960*,2961*,2964-29689 | F30*,F31 |
| Depression | 2962-29636, 3004,311 | F32*, F33* |
| *Telemedicine codes* |  |  |
| Telemedicine Codes without modifiers (HCPCS) | G0406, G0407, G0408, G0425,G0426,G0427, Q3014,G0459 | |
| Telemedicine Codes with modifiers (CPT) | 90791,90792, 90801, 90802, 90804, 90805,90806, 90807,90808, 90809, 90832, 90833, 90834, 90836, 90837, 90838, 90862, 90951, 90952, 90954, 90955, 90957, 90958, 90960, 90961, 92507, 92508, 92521, 92522, 92523, 92524, 97150,97165, 97166, 97167, 97168, 97530, 97802, 97803, 97804, 99201, 99202, 99203, 99204, 99205, 99211, 99212, 99213, 99214, 99215, 99241, 99242, 99243, 99244, 99245, 99251, 99252, 99253, 99254, 99255,99354, 99355, 99356, 99357, M0064, S9152, S9470  Modifier code of GT or 95 required for each CPT code | |

Notes: * displays that for each diagnosis code, the existing codes below each main code group are also included in the study.

**Appendix Table 2 – All Clients, Mental Health Clients, Tele Clients, and Mental Health Tele Clients**

| **All Clients** | |  |  |  | **Mental Health Clients** | | |  |  |
| --- | --- | --- | --- | --- | --- | --- | --- | --- | --- |
|  | **Total** | **Urban** | **Suburban** | **Rural** |  | **Total** | **Urban** | **Suburban** | **Rural** |
| 2013 | 54,455 | 13,634 | 32,375 | 8,446 | 2013 | 25,177 | 7,784 | 13,530 | 3,863 |
| 2014 | 65,844 | 21,286 | 35,899 | 8,659 | 2014 | 28,444 | 9,210 | 15,561 | 3,673 |
| 2015 | 78,909 | 31,462 | 40,426 | 7,021 | 2015 | 31,478 | 12,280 | 15,359 | 3,839 |
| 2016 | 87,565 | 29,879 | 49,908 | 7,778 | 2016 | 31,411 | 11,746 | 15,959 | 3,706 |
| 2017 | 106,101 | 42,585 | 53,144 | 10,372 | 2017 | 43,692 | 19,263 | 19,914 | 4,515 |
| 2018 | 126,580 | 48,419 | 66,984 | 11,177 | 2018 | 47,719 | 19,630 | 23,252 | 4,837 |
| Growth | 132.4% | 255.1% | 106.9% | 32.3% | Growth | 89.5% | 152.2% | 71.9% | 25.2% |
|  |  |  |  |  |  |  |  |  |  |
| **Telemedicine Clients** | |  |  |  | **Mental Health Telemedicine Clients** | | |  |  |
|  | **Total** | **Urban** | **Suburban** | **Rural** |  | **Total** | **Urban** | **Suburban** | **Rural** |
| 2013 | 5,596 | 1,383 | 3,703 | 510 | 2013 | 4,582 | 1,153 | 3,013 | 416 |
| 2014 | 6,244 | 2,365 | 3,249 | 630 | 2014 | 5,333 | 2,163 | 2,728 | 442 |
| 2015 | 7,741 | 2,648 | 3,761 | 1,332 | 2015 | 6,367 | 2,047 | 3,235 | 1,085 |
| 2016 | 8,818 | 2,472 | 5,151 | 1,195 | 2016 | 7,317 | 2,022 | 4,368 | 927 |
| 2017 | 9,480 | 3,576 | 4,732 | 1,172 | 2017 | 7,807 | 3,029 | 3,870 | 908 |
| 2018 | 10,145 | 3,496 | 5,214 | 1,435 | 2018 | 8,138 | 2,944 | 4,114 | 1,080 |
| Growth | 81.3% | 152.8% | 40.8% | 181.4% | Growth | 77.6% | 155.3% | 36.5% | 159.6% |

Notes: Each cell represents a unique set of disabled Medicaid clients who had a health care visit with a primary diagnosis of a mental health condition, a telemedicine visits, or had a telemedicine visit with a primary diagnosis of a mental health condition.

**Appendix Table 3 – Ordinary Least Squares Regression Results for All Clients, Mental Health Clients, Tele Clients, and Mental Health Tele Clients Growth Rate from 2013-2018**

| Growth Rate Clients | Mental Health | Telemedicine | Mental Health Telemedicine |
| --- | --- | --- | --- |
| Urban | 0.106 | -0.344 | -0.416 |
| Suburban | -0.336 | -0.463 | -0.439 |
| Cohort | 0.388 | 0.989† | 0.745 |
| Urban *Cohort | -1.316† | -0.325 | -0.239 |
| Suburban * Cohort | -0.994† | -0.488 | -0.392 |
| Age | -0.063 | -0.058 | 0.063 |
| Women | 17.615 | 12.923 | 6.682 |
| Non-Hispanic White | -3.200 | -3.245 | 6.131 |
| Non-Hispanic Black | 6.560 | 7.861 | 5.490 |
| Hispanic | -2.275 | -0.284 | 3.372 |
| Medicaid FFS | 0.029 | 0.127 | 0.997 |
| Dual eligible | -0.630 | 2.444 | 3.331 |
| Elixhauser Comorbidity Score | 0.049 | 0.025 | 0.657 |

Notes: Each column represents ordinary least squares regression results on the dependent variable of annual growth rates of patients with a mental health diagnosis vs without a mental health diagnosis, telemedicine vs non-telemedicine, and telemedicine visit for those patients with a mental health diagnosis vs those without telemedicine use. Cohort refers to the indicator variable identifying the group in each column header. Geographic references group is rural. † indicates statistically significant coefficient at the 1% level.

**Appendix Table 4 – Average Annual Mental Health Visits per Client Among Mental Health Clients**

| Average mental health visits  per mental health client | | | | | Average mental health visits  per mental health non-telemedicine client | | | | |
| --- | --- | --- | --- | --- | --- | --- | --- | --- | --- |
|  | Total | Urban | Suburban | Rural |  | Total | Urban | Suburban | Rural |
| 2013 | 14.3 | 14.3 | 13.9 | 15.4 |  | 13.7 | 13.8 | 13 | 15.4 |
| 2014 | 14.1 | 13.2 | 14.5 | 14.4 |  | 13.5 | 12.1 | 14 | 14.6 |
| 2015 | 14.0 | 13.6 | 14.0 | 15.0 |  | 13.6 | 13.1 | 13.8 | 14.6 |
| 2016 | 13.8 | 13.8 | 13.4 | 15.2 |  | 13.1 | 12.8 | 12.8 | 15 |
| 2017 | 15.2 | 13.5 | 16.6 | 16.7 |  | 14.8 | 12.9 | 16.4 | 16.5 |
| 2018 | 15.8 | 15.0 | 15.8 | 19.1 |  | 15.5 | 14.4 | 15.7 | 19.3 |
| Mean | 14.5 | 13.9 | 14.7 | 16.0 |  | 14 | 13.2 | 14.3 | 15.9 |
| Average mental health visits  per mental health telemedicine client | | | | | Average mental health tele visits  per mental health telemedicine client | | | | |
|  | Total | Urban | Suburban | Rural |  | Total | Urban | Suburban | Rural |
| 2013 | 16.9 | 17.2 | 17 | 16 |  | 2 | 2.1 | 1.9 | 2.1 |
| 2014 | 16.7 | 17.1 | 16.9 | 13.2 |  | 2 | 2.1 | 1.9 | 2.2 |
| 2015 | 15.4 | 16.1 | 14.7 | 16.1 |  | 2 | 1.9 | 1.9 | 2.3 |
| 2016 | 16.2 | 18.6 | 15.1 | 15.8 |  | 2 | 2.1 | 1.9 | 2.4 |
| 2017 | 16.9 | 16.5 | 17.1 | 17.3 |  | 2.1 | 2.1 | 2.2 | 2.1 |
| 2018 | 17.3 | 18 | 16.4 | 18.6 |  | 2.2 | 2.1 | 2.2 | 2.2 |
| Mean | 16.6 | 17.3 | 16.2 | 16.2 |  | 2 | 2.1 | 2 | 2.2 |

Notes: Each cell represents an average count of mental health visits per year stratified by telemedicine and non-telemedicine visits for different subgroups of mental health clients.

**Appendix Table 5 – Ordinary Least Squares Multivariate Regression results for All Clients, Mental Health Clients, Tele Clients, and Mental Health Tele Clients Growth Rate from 2013-2018**

| Growth Rate Clients | Mental Health | Telemedicine | Mental Health Telemedicine |
| --- | --- | --- | --- |
| Urban | 0.258 | -0.142 | -0.183 |
| Suburban | -0.256 | -0.309 | -0.314 |
| Cohort | 0.633 | 0.878† | 0.801 |
| Urban *Cohort | -1.238† | -0.331 | -0.260 |
| Suburban * Cohort | -0.862† | -0.446 | -0.344 |
| Age | -0.073 | -0.072 | -0.078 |
| Women | 17.045 | 12.849 | 8.364 |
| Non-Hispanic White | -3.936 | -3.254 | -4.424 |
| Non-Hispanic Black | 3.940 | 5.710 | 8.180 |
| Hispanic | -2.835 | -0.178 | 1.172 |
| Medicaid FFS | 0.012 | 0.070 | 0.160 |
| Dual eligible | -0.788 | 1.883 | 6.017 |
| Elixhauser Comorbidity Score | 0.094 | 0.183 | -0.113 |

Notes: Each column represents regression results on the dependent variable of annual growth rates of patient visits per person with a mental health diagnosis vs without a mental health diagnosis, telemedicine vs non-telemedicine, and telemedicine visit for those patients with a mental health diagnosis vs those without telemedicine use. Cohort refers to the indicator variable identifying the group in each column header. † indicates statistically significant coefficient at the 1% level.
